# Expression Levels of IL-17A, IL-17F, IL-17RA, and IL-17RC in Prostate Cancer with Taking into Account the Histological Grade according to Gleason Scale in Comparison to Benign Prostatic Hyperplasia: In Search of New Therapeutic Options

**DOI:** 10.1155/2020/4910595

**Published:** 2020-05-25

**Authors:** Marlena Janiczek, Łukasz Szylberg, Paulina Antosik, Anna Kasperska, Andrzej Marszałek

**Affiliations:** ^1^Department of Clinical Oncology, Greater Poland Cancer Center, Poznan, Poland; ^2^Department of Clinical Pathomorphology, Collegium Medicum in Bydgoszcz, Nicolaus Copernicus University, Torun, Poland; ^3^Department of Tumor Pathology and Pathomorphology, Oncology Center, Prof. Franciszek Łukaszczyk Memorial Hospital, Bydgoszcz, Poland; ^4^Department of Clinical Pathology, Poznan University of Medical Sciences and Greater Poland Cancer Center, Poznan, Poland

## Abstract

Prostate cancer (PCa) is the second most commonly diagnosed malignant tumor and the fifth leading cause of cancer death in men in the world. The most common types of tumors are adenocarcinomas. Prostate cancer is a slow-growing cancer. The incidence increases with age. Evaluation of proinflammatory factors such as IL-17A, IL-17F, IL-17RA, and IL-17RC expression makes it possible to assess the impact of inflammatory process on progression of PCa. The aim of the study was to retrospectively assess the histological material of PCa divided into few groups using the Gleason score. Studies were carried out on archival tissue material in the form of paraffin blocks of 40 men with PCa after radical prostatectomy. The control group was composed of 10 men with benign prostatic hyperplasia (BPH). The material was obtained by the transurethral resection of the prostate (TURP). Immunohistochemistry was performed on prepared material using specific primary antibodies against IL-17A, IL-17F, IL-17RA, and IL-17RC. Expression of the antibody to be examined using light microscopy and the Remmele-Stegner score (IRS) in cancer staining was then evaluated. Expression of IL-17 RA was not shown in a group of patients with PCa and in the control group. In the group of patients with Gleason score 8 and 9 PCa, the expression of IL-17A was higher compared to that of IL-17F. In addition, in PCa with an increased grade of Gleason scale, a decrease in the expression of the study inflammatory parameters was found. The inflammatory process has an impact on PCa. A study on IL-17 may become a starting point for further research on an attempt to use, for example, immunotherapy in PCa.

## 1. Introduction

Benign prostatic hyperplasia (BPH) and prostate cancer (PCa) are the most common urological diseases among older men [1]. PCa is the second most commonly diagnosed cancer and the fifth leading cause of cancer death worldwide [2]. In North America, more than 230,000 new cases of PCa and over 30,000 deaths have been reported in the year 2018 [3]. The incidence rates of these diseases increase with age. PCa develops slowly, and often, it is asymptomatic. Symptoms appear generally only in the advanced stages of the disease. The first symptoms may be from the metastases of PCa [4]. PCa remains one of the most curable malignancies, if it is detected early. However, in late-stage disease, the tumors become castration-resistant (CRPC) and become a deadly disease. Poor survival results in patients with metastatic CRPC influence the increase search of new therapeutic strategies [5]. In search of the new therapeutic strategies in PCa, inflammation should also be considered. A lot of data about the importance of inflammation for the development of prostate cancer microenvironment has been demonstrated.

Many cancers arise from the area of infection, chronic irritation, and inflammation. Inflammatory process is as element of neoplastic process, promoting proliferation, tumor cell survival, and migration [6, 7]. More and more evidence indicated chronic inflammation as a factor contributing to the development of PCa and progression to a metastatic disease. A recent meta-analysis showed that prostatitis and sexually transmitted infections could be correlated with an increased risk of PCa [8]. Furthermore, taking anti-inflammatory drugs and antioxidants was associated with a reduction in the risk of PCa [9]. Development of BPH is also connected with inflammation [10]. Chronic inflammation in BPH is associated with high-grade PCa [11]. Inflammation is a complex response involving many immune cells, chemokines, and cytokines as well as enzymes [12].

Interleukin-17 (IL-17, also called IL-17A) is a key proinflammatory cytokine. So far, IL-17 has been shown to promote the development of colon cancer, breast cancer, lung cancer, pancreatic cancer, and PCa [13]. IL-17 is secreted by helper T cell 17 (TH17), *γδ* T cells, NK cells, and other immune cells. IL-17 acts on the IL-17RA or IL-17RC receptor complex. IL-17 promotes the development of cancer through increased cell proliferation, attenuated apoptosis, and increased angiogenesis, as well as the formation of an immunotolerant microenvironment [12]. Studies suggest that the family of IL-17 may be associated with both BPH and PCa. Therefore, in our research, we wanted to examine the expression levels of IL-17A, IL-17F, and their receptors in PCa taking into account histological grades in the Gleason score and BPH. Currently, we have not found this type of research in the literature.

## 2. Material

Tissue material was selected from a group of 116 patients from years 2010-2017 after radical prostatectomy. Patients' age ranged from 50 to 76 years, and the mean age of patients was 67 years. The control group was composed of 10 men with BPH. The material was obtained using the transurethral resection of the prostate (TURP).

## 3. Method

The whole material was fixed in 10% buffered formalin and processed according to a standard protocol. Finally, paraffin blocks were prepared. The TMA Master obtained tissue microarrays from paraffin blocks. After preliminary evaluation of hematoxylin and eosin slides, the material was selected for immunohistochemical studies. We used primary antibodies against IL-17A (Sigma-Aldrich, HPA 052258, 1 : 175), IL-17F (Abcam, Ab 190340, 1 : 100), IL-17RA (Abcam, Ab 180904, 1 : 100), and IL-17RC (Abcam, Ab 69673, 1 : 100) and for detection–EnVision system (DAKO). Tonsil tissue was used as a positive control for IL-17A, IL-17RC, and IL-17RA. The large intestine was used as a positive control for IL-17F. Antigen expression evaluation in inflammatory infiltration of selected lesions was carried out using a modified Remmele-Stegner scale according to the intensity of expression and the number of positively expressed cells/tissue area (ranging from 1 (lowest expression) to 12 (highest expression)). The analysis was performed at 20x original objective magnification for each of the studied antibodies on 3 representative and randomly selected areas.

## 4. Statistics

The results were analysed statistically using the nonparametric Kruskal-Wallis test at a fixed level of significance of 0.05.

## 5. Ethical Issues

The study was carried out with the approval of the local ethics committee.

## 6. Results

### 6.1. IL-17A

**Figure 1:**
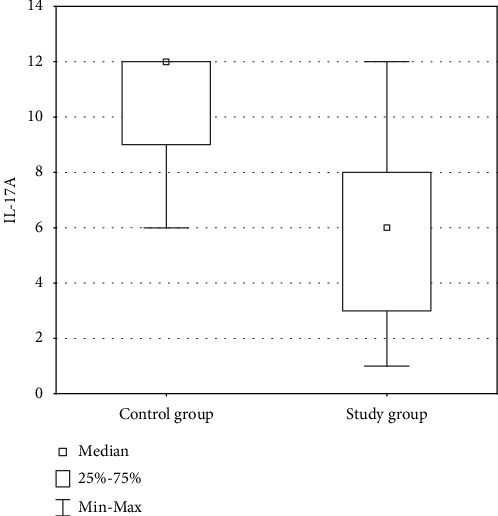
Comparison of expression level of IL-17A in the control group and the study group.

The cytoplasmic expression of IL-17A in PCa was found in 100% analysed cases of PCa. Higher levels of IL-17A were demonstrated in the control group compared to the study group, although not statistically significant (*p* > 0.05). The intensity of protein expression was diverse (from 1 to 12). The lowest expression was demonstrated in the group of patients with Gleason score 9 PCa. The highest expression was found in the control group (Figure 1).

### 6.2. IL-17F

**Figure 2:**
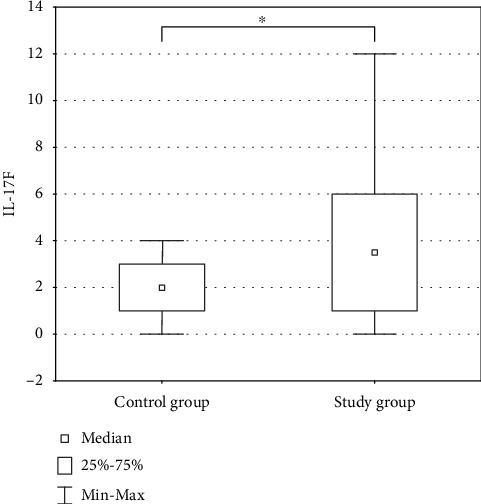
Comparison of expression level of IL-17F in the control group and the study group. Asterisks indicate the significance level between two groups (^∗^*p* < 0.05).

In the case of IL-17F, the presence of an immunohistochemical reaction product was demonstrated in the cytoplasm of the studied cells. The lowest expression was demonstrated in the control group of patients with BPH. The highest expression was shown in patients with Gleason score 6 PCa. A significant higher IL-17F expression was demonstrated in the study group in comparison to the control group (*p* < 0.05). In addition, it was shown that the expression of IL-17F was lower in PCa with higher histological grade (Gleason score > 7) (Figure 2).

## 7. The Level of Expression of IL-17A in Comparison to IL-17F

**Figure 3:**
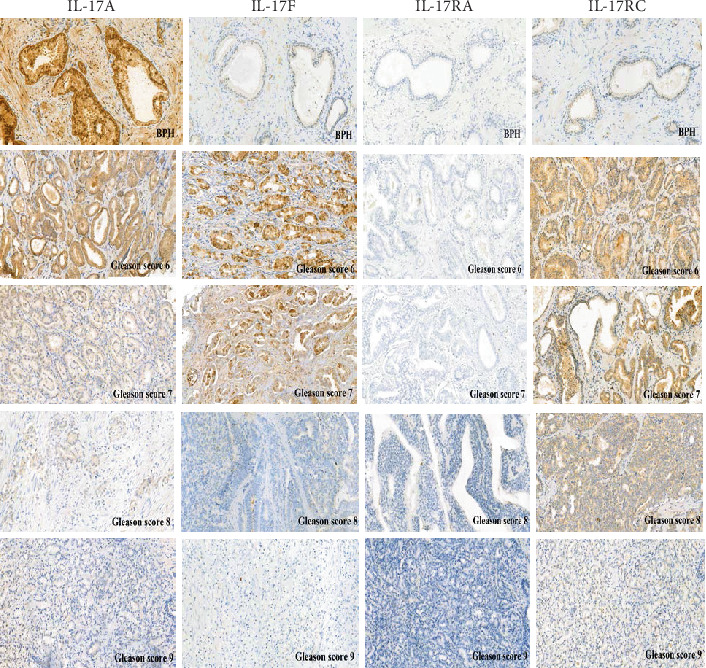
Expression of IL-17A, IL-17F, IL-17RA, and IL-17RC in prostate cancer divided into groups using the Gleason score and benign prostatic hyperplasia (BPH) (20x magnification).

Expression of IL-17A and IL-17F in the patients with Gleason score 6 and 7 PCa was comparable (not statistically significant, *p* > 0.05). In the group of patients with Gleason score 8 and 9 PCa, the expression of IL-17A was higher compared to that of IL-17F (Figure 3).

### 7.1. IL-17RA

Evaluation of the expression IL-17RA was performed in the cytoplasm and membrane of PCa cells. A small expression was found in several of the analysed cases of PCa and BPH (*p* < 0.05). There were no differences between the group with BPH and the study group.

### 7.2. IL-17RC

**Figure 4:**
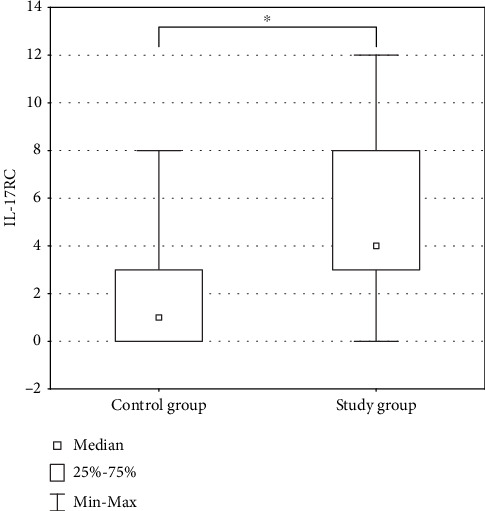
Comparison of expression level of IL-17RC in the control group and the study group. Asterisks indicate the significance level between two groups (^∗^*p* < 0.05).

In the case of IL-17RC, the expression was assessed on the basis of the presence of a colored reaction product in the cytoplasm and the membrane of the PCa cells studied. The level of this expression was diverse (from 0 to 12). The lowest expression was demonstrated in the control group of patients with BPH. The highest expression was demonstrated in patients with Gleason score 6 and 7 PCa. In addition, IL-17RC was found to be more highly expressed in the control group compared to the study group (*p* < 0.05). In addition, it was shown that the higher the histological grade of PC, the lower the expression of IL-17RC (Figure 4).

## 8. The Level of Expression of IL-17RA in Comparison to IL-17RC

The immunohistochemistry performed showed a higher level of IL-17RC as compared to IL-17RA in all histological grades of the Gleason score (Figure 3).

## 9. Discussion

Cancer is a chronic disease with a relatively large number of genes involved in pathogenesis [14]. Chronic inflammation contributes to the occurrence and progression of cancer in humans by modifying the tumor microenvironment. At the biological level, inflammatory cells release cytokines that act on tumor growth and metastasis. In prostate glands, inflammation correlates with development of lesions like proliferative inflammatory atrophy (PIA). PIA contains activated inflammatory cells and proliferating epithelial cells. PIA can be a pathological precursor to prostatic intraepithelial neoplasia (PIN) and/or PCa [15]. When PIN lesions were detected in biopsies, the chance of finding a cancer increases to 30%. High-grade PIN lesions occur in above 80% of patients with radical prostatectomy. Despite such a high incidence, it is hard to show the impact of prostatitis on the progression of PCa [16]. Moreover, it was shown that, in prostate biopsies, intraprostatitis is often detected in patients with elevated prostate-specific antigen (PSA) [17, 18]. These data suggestion that chronic inflammation in BPH is associated with high-grade PCa [11].

In recent years, both laboratory and clinical researches have revealed that immune escape is one of the most important mechanisms of cancer. This immune escape mechanism is used to avoid destruction by the human immune system and acquisition of resistance to anticancer drugs. This becomes a key barrier in the treatment of cancer. Many factors impact on the cancer mechanism of immune escape [19]. In recent years, therapeutic options to allow the immune system to come back to fight cancer have been introduced. These are intrinsic antibodies that inhibit immune checkpoints as well as T cells of the chimeric antigen receptor. After these early milestones in immunotherapy, there was a renewed interest in the antitumor properties of cytokines. This has led to a sharp increase in the number of clinical trials that investigate the safety and efficacy of cytokine-based drugs, as well as their combination with other immunomodulatory drugs. Cytokines are molecular messengers that allow cells in the immune system to communicate with each other in order to achieve a coordinated, robust but self-limiting response to the target antigen [20]. Recently, much research has been devoted to the relatively recently discovered IL-17 (also called IL-17A) [21].

Interleukin-17 is a key proinflammatory cytokine that has been shown to promote the development of cancers [22]. Recently, IL-17 was detected in colon cancer, lung cancer, bladder cancer, and PCa which aroused great interest among scientists. This caused a lot of controversy in terms of their functional impact and significance as prognostic biomarkers in different cancers [23]. However, much remains to be understood before we can consider IL-17 as a target for immune interventions in cancer therapy.

Many previous studies seem to prove that the IL-17 family are able to influence to development of BPH and PCa. However, currently, there is no systematic comparative study of expression levels of IL-17 and their respective receptors in PCa with taking into account the histological grade. We analysed cytokines of the IL-17 family, that is, IL17A and F, and their receptors IL-17RA and IL-17RC in prostate tissues in patients with BPH and PCa. At the same time, we examined the presence of these factors in PCa with taking into account the histological grade according to the Gleason scale.

Liu et al. have shown that, in obese mice, hyperinsulinemia enhanced IL-17-induced expression of downstream proinflammatory genes with increased levels of IL-17RA, resulting in development of more invasive PCa. Glycogen synthase kinase 3 (GSK3) was constitutively bound to and IL-17RA phosphorylation at T780. It was leading to ubiquitination and proteasome-mediated degradation of IL-17RA, thus inhibiting IL-17-mediated inflammation. In the proliferative PCa cells compared to the normal cells, IL-17RA phosphorylation was reduced, while the IL-17RA levels were increased. Insulin and IGF1 enhanced IL-17-induced inflammatory responses through suppressing GSK3. It was shown in the cultured cell lines in vitro and obese mouse models of PCa in vivo [24]. In another research, Liu et al. have demonstrated increased expression of IL-17A, IL-17RA, IL-17E, and IL-17F in the prostate gland both in BPH and in PCa compared to the expression in the control group. It was accompanied by increased numbers of infiltrating inflammatory cells. PCa was characterized by reduced immunoreactivity for IL-17BR and reduced numbers of CD68+ macrophages, fibroblasts, and smooth muscle cells, compared with BPH. It was a trend for these changes to correlate with severity of disease in both PCa and BPH. These data showed that IL-17A acting through IL-17RA, but not IL-17CR, contributed to the pathogenesis of BPH and PCa. In contrast, IL-17E interacting with the IL-17BR might have an antitumor effect [25]. Our study has not confirmed this hypothesis. In our studies, we have shown no expression of IL-17RA in both BPH and PCa (with taking into account all histological grades according to the Gleason scale). We have not found reference to histological grades in currently available researches; our research is the first of this type regarding IL-17RA. In addition, our studies have confirmed the presence of IL-17A and IL-17F in the development of BPH and PCa. We have shown that the IL-17A and IL-17F expression levels were higher in PCa with a histological grade in Gleason scores 6 and 7. Moreover, we have shown that in the group of patients with Gleason score 8 and 9 PCa, the expression of IL-17A was higher compared to that of IL-17F.

Another study on IL-17 was presented by Zhang et al. They found that there was a reduction in the formation of microinvasive PCa in PTEN-null mice treated with the antibody SR1001 or anti-IL-17. In addition, mouse prostates treated with SR1001 or anti-IL-17 antibody have reduced proliferation and angiogenesis, as well as reduced infiltration of inflammatory cells and increased apoptosis. In addition, they found that SR1001 or anti-IL-17 antibody-treated prostate tissues had weaker EMT phenotype compared to the control-treated prostates. It was assesses by the epithelial-to-mesenchymal transition (EMT) markers [26]. In subsequent studies, Zhang et al. investigated the genetic approach in PCa. There was interbreeding of IL-17RC-deficient mice with mice that are conditionally mutant for PTEN. It was one established preclinical model for PCa. Mice that were IL-17RC-deficient (IL-17RC(-)) displayed prostates that were smaller than those of mice that maintained IL-17RC expression (IL-17RC(+)). Moreover, IL-17RC(+) mice developed an increased number of invasive prostate adenocarcinomas with higher rates of cellular proliferation and lower apoptosis than IL-17RC(-) mice. In addition, the fibromuscular stroma surrounding prostatic glands was relatively thicker in IL-17RC(-) mice and was associated with decreased matrix metalloproteinase (Mmp)7 expression and increased Timp1, 2, and 4 expression, whereas administration of recombinant mouse IL-17 induced prostatic expression of Mmp7 [27]. In the other research, Zhang et al. have shown that RC(-) mouse prostates were smaller than those of RC(+) mice. Approximately 23% of prostatic glands in RC(-) mice, in contrast to 65% of prostatic glands in RC(+) mice, developed invasive adenocarcinomas. Castrate RC(-) mouse prostate had lower rates of cellular proliferation and higher rates of apoptosis as well as lower levels of MMP7, YBX1, MTA1, and UBE2C proteins, compared to castrate RC(+) mice. In addition, castrate RC(-) mouse prostate had less angiogenesis. It was associated with decreased levels of COX-2 and VEGF. In addition, castrate RC(-) mouse prostate had fewer inflammatory cells, i.e., lymphocytes, macrophages, and myeloid-derived suppressor cells. These data suggest that IL-17 promotes the development of invasive PCa under castration conditions, potentially by creating an immunotolerant and proangiogenic tumor microenvironment [28]. You et al. have shown that IL-17RC have few different isoforms. The isoform as detected by anti-IL-17RC intracellular domain antibodies (anti-ICD) was expressed at higher levels in androgen-independent prostate cancer cell lines (PC3 and DU145) than in androgen-dependent prostatic cell lines (RWPE-1, pRNS-1-1, MLC-SV40, and LNCaP). Moreover, several isoforms as detected by anti-IL-17RC extracellular domain antibodies (anti-ECD) were expressed at significantly higher levels in androgen-dependent prostatic cell lines than in androgen-independent ones. IL-17RC protein expression was significantly higher in androgen-independent PCa than in androgen-dependent ones when anti-ICD was used. And when anti-ECD was used, IL-17RC protein expression was significantly higher in androgen-dependent PCa than in androgen-independent ones. These observations provide evidence that IL-17RC protein isoforms are diversified in normal prostate and tumor cells and may play a negative or positive role in PCa initiation and progression [29]. In our study, we also demonstrated the expression in IL-17RC in BPH and PCa. The lowest expression was demonstrated in the control group of patients with BPH. The highest expression was demonstrated in patients with Gleason score 6 and 7 PCa. These data show that IL-17RC is bound mainly with lower histological grade of PCa.

In another study, Vykhovanets et al. have demonstrated the association of IL-17-producing mononuclear cell formation in areas of PCa, PIA, or BPH. In addition, levels of IL-17-producing cells were shown to be similar in the BPH and PCa areas. A clear accumulation of IL-17 cells in the lumen and around the gland was identified in mononuclear cell infiltrates associated with PIA lesions. Glandular and around glandular macrophages and CD68+ neutrophils were the dominant IL-17-producing cells in PIA lesions. The accumulation of cells expressing IL-17 in PIA lesions can be evidence of an inflammatory microenvironment that can support the development of PCa [30].

In our study, we have shown that IL-17A and IL-17F act through IL-17RC, but not IL-17RA. These inflammatory factors contribute to the pathogenesis of BPH and PCa. In addition, we have shown that the IL-17A, IL-17F, and IL-17RC expression levels were higher in lower grade PCa, i.e., histological grade in Gleason scores 6 and 7. These data also suggest that patients with prostate cancer with increased expression of IL-17A, IL-17F, and IL-17RC have a better prognosis than patients whose cancer cells do not express these interleukins. We have not currently found this type of research taking into account the histological grades in PCa.

## 10. Conclusion

The data presented above are evidence of huge efforts to understand the tumor inflammatory microenvironment as a basic element to create an effective prevention or treatment of PCa. These data also confirm that the role of IL-17 and its receptors in the carcinogenesis of PCa is still not clearly defined. In our study, we have not demonstrated the presence of IL-17RA in BPH and in PCa in any case, regardless of the Gleason grade. Moreover, we have shown that IL-17A and IL-17F acting through IL-17RC contributed to the pathogenesis of BPH and PCa. In addition, we have demonstrated that in PCa, in a higher histological grade, expression of IL-17F was more frequently demonstrated compared to that of IL-17A. Based on the research, it can be concluded that the inflammatory process is more intense in BPH compared to PCa. In addition, there is a correlation between the histological grade of the Gleason score and the level of expression of the assessed parameters of inflammation. The lower the histological grade of the Gleason score of PCa, the higher the expression of selected factors of the inflammatory process. These data suggest that the inflammatory process is more important in the development of a lower grade of PCa. Our results may be useful in better understanding of PCa pathogenesis and in predicting its clinical course. We hope that the results of our research will also be able to serve as an element in planning for further research on immunotherapy. In current data, the role of IL-17 is ambiguous. In connection, the research on IL-17 should be continued in order to precisely establish the role of this cytokine in the inflammatory process involved in the development of PCa.

## Data Availability

Currently, I cannot share data, because I plan to publish a series of publications on the basis of all the data.
